# Is the New Primate Genus *Rungwecebus* a Baboon?

**DOI:** 10.1371/journal.pone.0004859

**Published:** 2009-03-19

**Authors:** Dietmar Zinner, Michael L. Arnold, Christian Roos

**Affiliations:** 1 Cognitive Ethology, Deutsches Primatenzentrum, Göttingen, Germany; 2 Department of Genetics, University of Georgia, Athens, Georgia, United States of America; 3 Primate Genetics and Gene Bank of Primates, Deutsches Primatenzentrum, Göttingen, Germany; Ecole Normale Supérieure de Lyon, France

## Abstract

**Background:**

In 2005, a new primate species from Tanzania, the kipunji, was described and recognized as a member of the mangabey genus *Lophocebus*. However, molecular investigations based upon a number of papionins, including a limited sample of baboons of mainly unknown geographic origin, identified the kipunji as a sister taxon to *Papio* and not as a member of *Lophocebus*. Accordingly, the kipunji was separated into its own monotypic genus, *Rungwecebus*.

**Methodology/Principal Findings:**

We compare available mitochondrial and nuclear sequence data from the voucher specimen of *Rungwecebus* to other papionin lineages, including a set of geographically proximal (parapatric) baboon samples. Based on mitochondrial sequence data the kipunji clusters with baboon lineages that lie nearest to it geographically, i.e. populations of yellow and chacma baboons from south-eastern Africa, and thus does not represent a sister taxon to *Papio*. Nuclear data support a *Papio*+*Rungwecebus* clade, but it remains questionable whether *Rungwecebus* represents a sister taxon to *Papio*, or whether it is nested within the genus as depicted by the mitochondrial phylogeny.

**Conclusions/Significance:**

Our study clearly supports a close relationship between *Rungwecebus* and *Papio* and might indicate that the kipunji is congeneric with baboon species. However, due to its morphological and ecological uniqueness *Rungwecebus* more likely represents a sister lineage to *Papio* and experienced later introgressive hybridization. Presumably, male (proto-)kipunjis reproduced with sympatric female baboons. Subsequent backcrossing of the hybrids with kipunjis would have resulted in a population with a nuclear kipunji genome, but which retained the yellow/chacma baboon mitochondrial genome. Since only one kipunji specimen was studied, it remains unclear whether all members of the new genus have been impacted by intergeneric introgression or rather only some populations. Further studies with additional *Rungwecebus* samples are necessary to elucidate the complete evolutionary history of this newly-described primate genus.

## Introduction

In 2005, a new primate species from Tanzania, the kipunji, was described and originally recognized as a member of the mangabey genus *Lophocebus*, mainly based on its arboreality and non-contrasting black eyelids [1]. However, subsequent molecular studies suggested that the kipunji was more closely related to *Papio*, rather than to one of the two mangabey genera or any other member of the Papionini tribe [2]. Based on these findings, the kipunji was recently placed into its own genus, *Rungwecebus* [2]. Besides genetic evidence, the uniqueness of *Rungwecebus* was also supported by morphological, acoustic, behavioral and ecological characteristics [2]–[4]. Nevertheless, the classification of *Rungwecebus* as a new genus has been questioned [5].

**Figure 1:**
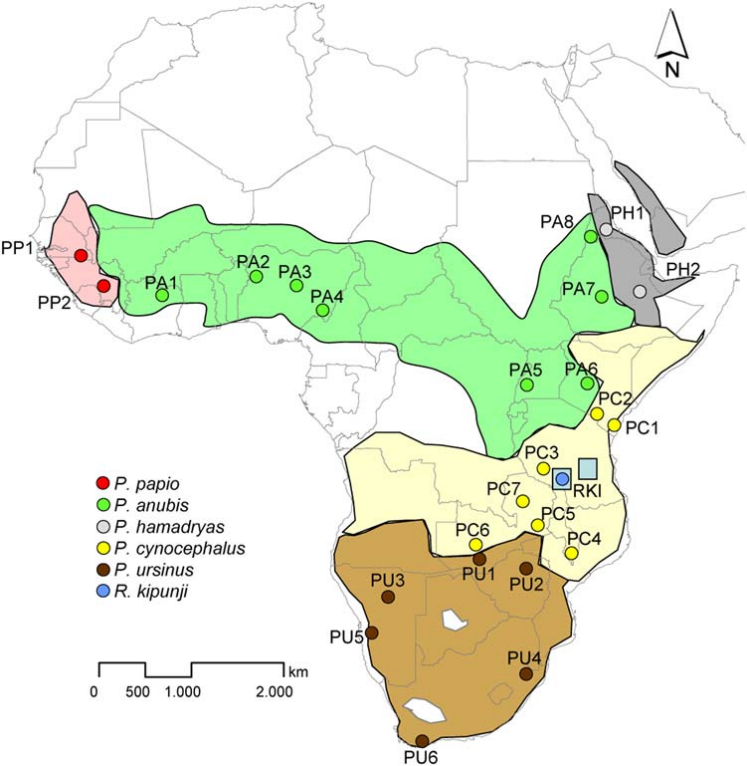
Distribution of *Papio* [modified after 63] and *Rungwecebus* (blue boxes), and collecting sites of samples. Blue dot = origin of the *Rungwecebus* sample [2] from the Southern Highlands of Tanzania. A second population of *Rungwecebus* was found in the Udzungwa Mountains [1]. Names of baboon collecting sites and their geographical coordinates are given in Table S1.

To place *Rungwecebus kipunji* phylogenetically, Davenport et al. [2] generated sequences of three mitochondrial (cytochrome oxidase subunit I, COI; cytochrome oxidase subunit II, COII; 12SrRNA) and two nuclear (α 1,3 galactosyltransferase, α 1,3-GT; Y chromosomal testis-specific protein, TSPY) loci of a single voucher specimen. In 2008, another three nuclear loci (autosomal lipoprotein, LPA; autosomal gene encoding CD4; X chromosomal region, Xq13.3) of *Rungwecebus* became available and the TSPY sequence data were expanded [6]. In both studies, the *Rungwecebus* data were compared with orthologous sequences of other Old World monkeys, deposited in GenBank. However, the GenBank data did not cover the full taxonomic and geographic range of *Papio* (Figure 1). In particular, sequences from *Papio* of southern Tanzania and neighboring regions, those geographically nearest to the range of *Rungwecebus*, were not available for both analyses [2], [6]. Furthermore, many of the *Papio* sequences in GenBank are of unknown geographic origin because they derived from captive animals. In the course of a study on the evolution and phylogeography of *Papio*, we compared the available nuclear and mitochondrial sequence data of *Rungwecebus* with orthologous data of baboons from known geographic origin and representatives of all other papionin genera.

## Results

The main result of our study is that, based on mitochondrial sequence variation, *Rungwecebus* does not constitute a sister taxon to *Papio* but clusters within *Papio*. This result contrasts with those of both Davenport et al. [2] and Olson et al. [6]. However, this relationship becomes obvious only when baboons from parapatric populations to *Rungwecebus* from south-east Africa are included in the study. The analysis of nuclear data provides limited power due to the extremely low levels of variation and hence, the relationships among baboons and the kipunji cannot be resolved unambiguously using these data.

**Figure 2:**
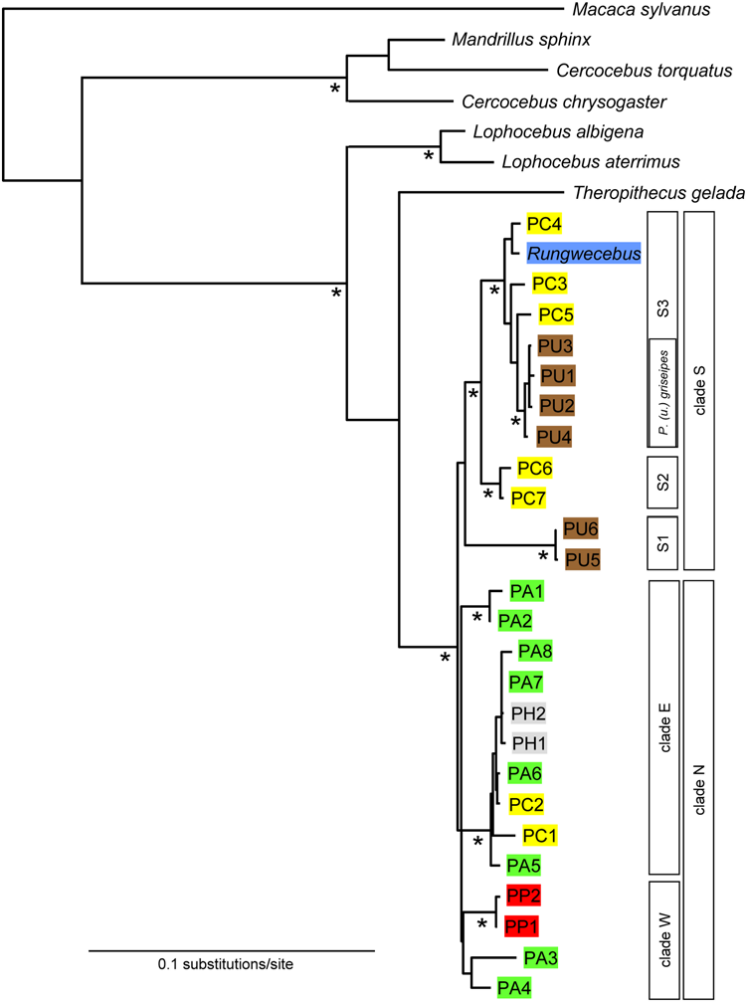
Phylogenetic position of *Rungwecebus* in relation to *Papio* and other members of the Papionini - mitochondrial DNA phylogeny. The phylogram is based on the neighbor-joining algorithm and by applying the TrN+I+G model of sequence evolution. Marked * nodes yielded bootstrap values of ≥85% (for MP, NJ and ML) or posterior probability values of ≥0.95 (Bayesian). Red = *P. papio*, green = *P. anubis*, grey = *P. hamadryas*, yellow = *P. cynocephalus*, brown = *P. ursinus*. Bars on the right side of the phylogram denote respective baboon clades and are discussed in the text.

Our mitochondrial data sets included all seven papionin genera. Furthermore, the 25 baboon individuals represented all five *Papio* species (*P. papio*, *P. hamadryas*, *P. anubis*, *P. cynocephalus*, *P. ursinus*) and covered most of the genus' geographic distribution. Phylogenetic tree reconstructions for the three individual loci (Figures S1, S2, S3) and the concatenated data set with 1486 bp in length (Figure 2) revealed mainly identical and highly supported relationships, with only a few remaining unresolved or with low support. We found strong support for the division of African papionins into two major clades, one with *Mandrillus* and *Cercocebus*, and the other with *Lophocebus*, *Theropithecus*, *Papio* and *Rungwecebus*. Among the latter, a common origin of *Papio* and *Rungwecebus* was highly supported, but the relationship between this clade and either *Lophocebus* or *Theropithecus* was not well resolved. Within the *Papio*+*Rungwecebus* clade, we found several strongly supported haplogroups. However, these did not correspond to the traditionally recognized baboon species, and with the exception of *P. papio*, all other baboon taxa were para- or polyphyletic. In contrast, we found a strong geographical signal with local populations forming monophyletic haplogroups irrespective of their species affiliations. This reflects clearly the discordance between mitochondrial phylogeny and baboon morphology. The same is also true for *Rungwecebus*, which did not represent a sister lineage to the *Papio* genus [2], [6], but instead clustered with yellow baboons (*P. cynocephalus*) from southern Tanzania, Malawi, Zambia and with chacma baboons (*P. ursinus*) from Zimbabwe, northern Namibia and northern South Africa. These yellow and chacma baboons from south-east Africa represent local populations that are geographically closest to *Rungwecebus* (Figure 1). To test for the reliability of the depicted relationships, we evaluated alternative phylogenetic positions of *Rungwecebus* among papionins with the Kishino-Hasegawa (KH) [7] and Shimodaira-Hasegawa (SH) [8] tests. Accordingly, a sister grouping of *Rungwecebus* to *Papio*, *Theropithecus*, *Lophocebus* or a *Cercocebus*+*Mandrillus* clade was significantly rejected (P<0.001, Table 1).

**Table 1:** Log likelihoods for alternative tree topologies.

tree topology	mitochondrial DNA		nuclear DNA	
	- Log Likelihood	KH/SH test	- Log Likelihood	KH/SH test
*Rungwecebus* clusters within *Papio*	5653.26440	**best tree**	7373.63688	P = 0.578; P = 0.604
*Rungwecebus* sister lineage to *Papio*	5728.00367	P<0.001	7369.50337	**best tree**
*Rungwecebus* sister lineage to *Theropithecus*	5736.60755	P<0.001	7416.41553	P<0.05
*Rungwecebus* sister lineage to *Lophocebus*	5738.32191	P<0.001	7416.41553	P<0.05
*Rungwecebus* sister lineage to (*Mandrillus*+*Cercocebus*)	5750.55459	P<0.001	7490.88793	P<0.001

**Figure 3:**
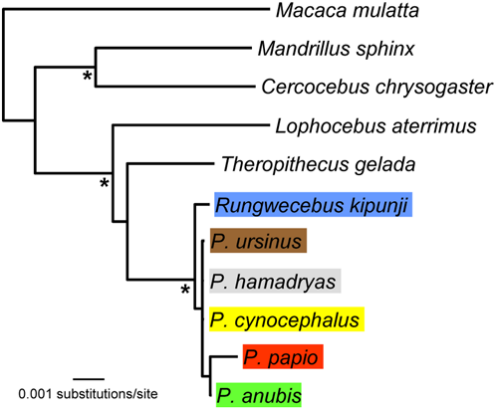
Phylogenetic position of *Rungwecebus* in relation to *Papio* and other members of the Papionini - nuclear DNA phylogeny. The phylogram is based on the neighbor-joining algorithm and by applying the TrN+I model of sequence evolution. Marked * nodes yielded bootstrap values of ≥98% (for MP, NJ and ML) or posterior probability values of 1.0 (Bayesian). For abbreviations and geographic origin of baboon sequences see Figure 1 and Table S1.

Each of our nuclear data sets comprised 11 sequences, of which five were derived from the five baboon species and the remaining six from representatives of the other papionin genera. Phylogenetic tree reconstructions for individual loci (Figures S4, S5, S6, S7, S8) and the concatenated data set of 4486 bp in length (Figure 3) provided similar tree topologies, although the resolution was relatively low, especially for single loci. As in the mitochondrial phylogeny, the nuclear data strongly supported a major division of African papionins into a *Cercocebus*+*Mandrillus* and a *Lophocebus*+*Theropithecus*+*Papio*+*Rungwecebus* clade. Among the latter, the clustering of *Papio* and *Rungwecebus* was significant. However, due to the low number of polymorphic sites (Table S4) we were unable to resolve the relationships among the five baboon species and whether *Rungwecebus* is nested within the genus *Papio* or represents its sister lineage. Only one transversion (guanine present in all *Papio* species, thymine in all other papionin genera) at position 534 in the TSPY alignment provided some indications that *Rungwecebus* might be basal. Alternative tree topology tests significantly rejected a sister grouping of *Rungwecebus* to *Theropithecus*, *Lophocebus* or a *Cercocebus*+*Mandrillus* clade (P<0.05, P<0.001, Table 1). Although a tree with *Rungwecebus* as a sister lineage to *Papio* represents the most likely relationship, an unresolved polytomy including *Rungwecebus* and the five *Papio* species was not rejected (P = 0.578, P = 0.604, Table 1). Therefore, a phylogeny with *Rungwecebus* nested within the *Papio* clade cannot be excluded.

Due to the low number of variable sites in the nuclear data set, divergence ages were estimated only for mitochondrial data. Based on our estimates, the initial split separating southern baboon lineages+*Rungwecebus* (clade S) from northern baboon lineages (clade N) occurred 2.19 million years ago (mya) (95% confidence limit [CI]: 1.50–2.98 mya) (Table 2, Figure 2). Among northern baboons, western populations including *P. papio* and *P. anubis* (clade W) and eastern populations with *P. hamadryas*, *P. anubis* and *P. cynocephalus* (clade E) diverged from each other 1.69 (1.04–2.41) mya. The southern clade including *Rungwecebus* is further divided into three subgroups. *P. ursinus* (PU5, PU6) from the South and West of southern Africa (clade S1) diverged from the remaining southern populations 2.02 (1.37–2.79) mya and most likely represent Cape chacmas (*P. (u.) ursinus*). Afterwards, 1.36 (0.82–2.01) mya, *P. cynocephalus* from central Zambia (PC6, PC7; clade S2), which represent Kinda baboons (*P. (c.) kindae*) [9]–[11], were separated from additional populations of *P. cynocephalus* and *P. ursinus* as well as from *Rungwecebus* (clade S3). *Rungwecebus* diverged from its closest related baboon haplotype (PC4) 0.35 (0.09–0.67) mya. The distinct subclade of *P. ursinus* within clade S3 most likely represents grey-footed chacmas (*P. (u.) griseipes*) [9]–[11].

**Table 2:** Bayesian divergence date estimates in mya (C denotes calibration points).

	mean	95% credibility interval
*Homo*/*Pan* (C1)	6.48	6.10–6.87
Hominoidea/Cercopithecoidea (C3)	23.70	21.65–25.55
*Colobus*/Cercopithecinae	14.26	11.14–18.12
*Chlorocebus*/Papionini	10.20	9.27–11.09
*Macaca*/other Papionini	9.18	7.71–10.48
*Macaca mulatta/M. sylvanus*	5.86	3.59–8.00
(*Mandrillus*+*Cercocebus*)/(*Lophocebus*+*Theropithecus*+*Papio*+*Rungwecebus*)	8.47	6.98–10.00
*Mandrillus/Cercocebus*	3.73	2.09–5.68
*Lophocebus*/(*Theropithecus*+*Papio*+*Rungwecebus*)	4.95	3.79–6.15
*Theropithecus*/(*Papio*+*Rungwecebus*) (C2)	3.75	3.20–4.28
northern *Papio* (N)/(southern *Papio*+*Rungwecebus*) (S)	2.19	1.50–2.98
western (W)/eastern (E) *Papio*	1.69	1.04–2.41
*P. ursinus* (PU5,PU6) (S1)/other southern lineages (S2,S3)	2.02	1.37–2.79
*P. cynocephalus* (PC6,PC7) (S2)/other southern lineages (S3)	1.36	0.82–2.01
*Rungwecebus/P. cynocephalus* (haplotype PC4)	0.35	0.09–0.67

## Discussion

Based on its morphology [1], [5], the kipunji was first regarded as a mangabey of the genus *Lophocebus*. However, subsequent genetic, cranial morphometric and ecological analyses provided evidence for the uniqueness of the kipunji and led to its being placed into a new genus [2], [4], [6]. Moreover, the then available genetic data suggested a sister taxon relationship of *Rungwecebus* with *Papio* and not with *Lophocebus* or any other papionin genus [2], [6].

In contrast to Davenport et al. [2] and Olson et al. [6] we found no clear sister taxon relationship between the kipunji and the baboon clade. Instead, our analyses of mitochondrial sequences of the kipunji demonstrated that this lineage clusters closely with baboon lineages that lie nearest to it geographically (i.e. yellow baboons from southern Tanzania, Malawi and eastern Zambia and chacma baboons from Zambia, Zimbabwe, northern Namibia and northern South Africa). In contrast, nuclear sequence data give some indication that *Rungwecebus* is a sister taxon to *Papio* and not nested within the baboon clade. Although, this relationship is only weakly supported by the available nuclear data, morphological and ecological data clearly provide evidence for the distinctiveness of *Rungwecebus* and its separation from *Papio* [3], [4]. Likewise for *Papio*, discordance between the mitochondrial phylogeny and the traditional classification into five clearly differentiated species [12], [13] was found. Incomplete lineage sorting of mitochondrial DNA could be a possible explanation of such discrepancies, but then *Rungwecebus* would be expected to cluster with more ancient baboon lineages and not specifically with geographically-adjacent baboon haplotypes. The same is also true for baboon lineages, where local populations form monophyletic groups irrespective of their species affiliations. Hence, the observed discrepancy between mitochondrial data on the one hand and morphological, ecological (and nuclear) data on the other hand raises questions about the evolutionary history of *Rungwecebus* and *Papio*.

A possible explanation could be that *Rungwecebus* is actually a member of the genus *Papio* as suggested by the mitochondrial data and thus does not warrant placement into its own genus. In support of such a hypothesis, the mitochondrial sequence data yield a relatively recent divergence time (i.e. 0.35 [0.09–0.67] mya) between *Rungwecebus* and geographically-associated baboon lineages. If *Rungwecebus* belongs within *Papio*, its unique morphological and ecological characteristics, which are clearly different from that of baboons [2], [5], would reflect recently acquired autapomorphies. Kipunjis are more arboreal than baboons [2], [5] and hence, depend on woodland habitats. During the Pleistocene, climatic changes led to shifts in the distribution of forest habitats and savannah biotopes [14]–[18]. During such events baboon populations might have become isolated and adapted to forest or woodland habitats, resulting in a new and distinct morphotype, the kipunji. However, baboons living in forests in other parts of Africa (e.g. olive baboons in eastern and north-eastern Democratic Republic of Congo) did not adapt specifically to a more arboreal life [19].

A more likely explanation is that *Rungwecebus* is the result of introgressive hybridization. Hybridization and introgression events are well known for African papionins including baboons (within *Papio* [20]; between the genera *Theropithecus* and *Papio*, [21], [22]; reviewed by [23]). In fact, Figure 2 shows multiple para- and polyphylies of baboon species, which is consistent with previously-described introgressive hybridization and nuclear swamping events among various baboon lineages [e.g. 9]–[11], [ 24]–[28]. For a broader view of *Papio* phylogeny see [9]–[11].

Hybridization and introgression have been considered important in the generation of plant diversity, and an appreciation of their role in the evolutionary diversification of animals has been growing over the past decade [29]–[32]. Besides papionins, additional examples of hybridization between other primate species, and even genera, are well documented for a number of clades [reviewed in 23], [33]. Introgressive hybridization appears to have likely played a role in the evolution of hominoids, including *Homo sapiens* [23], [30], [33]–[36]. There is also evidence that *Trachypithecus pileatus*, *Macaca arctoides* and *Macaca munzala* are the products of hybridization or introgression [37]–[39].

In the case of the kipunji, introgression of the maternally-inherited mitochondrial DNA from baboons into *Rungwecebus* seems to be likely. A possible scenario that would explain the introgression detected by our study is that a small population of baboons became isolated within the range of kipunjis resulting in female baboons reproducing with male kipunjis until 0.35 (0.09–0.67) mya. As mentioned above, repeated shifts in the extent of forest and savannah habitats during the Pleistocene may have promoted the isolation of local baboon populations. Given backcrossing of the hybrid offspring with kipunji or proto-kipunji males, over several generations, the frequency of baboon nuclear genes within the hybrid population would have decreased sharply. The result of such a process would have been a population with an almost complete kipunji nuclear genome, but with a baboon mitochondrial genome. This is what we have detected in our analyses. Since morphological characteristics would most likely be determined by nuclear genes, members of such a population would be expected to resemble kipunjis rather than baboons.

Based on our findings, the newly described genus *Rungwecebus* might be congeneric with baboon species and thus, *Rungwecebus* would be synonymous with *Papio*. Alternatively, and we would argue more likely, *Rungwecebus* represents a sister lineage to *Papio* and experienced later introgressive hybridization. However, since only a single *Rungwecebus* individual from the population in the southern highlands was studied, it is not clear whether the entire kipunji population possesses 1) a *Papio*-like mitochondrial DNA in general or 2) an admixture of *Papio*-like and undefined *Rungwecebus* haplotypes. Regardless of whether or not the detected mitochondrial haplotype of *Rungwecebus* turns out to be characteristic for only the examined specimen (or for only a subsample of the individuals belonging to this species) the evolution of *Rungwecebus* has been reticulate. Defining the extent of the reticulate (i.e. introgressive hybridization) events will require further molecular studies that incorporate more individuals and additional mitochondrial and nuclear loci.

## Materials and Methods

### Ethics statement

Our work was conducted according to relevant German and international guidelines, including countries where we collected fecal samples. Fecal samples were collected in a non-invasive way without disturbing, threatening or harming the animals. Blood samples were taken from zoo animals by zoo veterinarians for diagnostic reasons to check the health status of the respective individuals. Blood samples were explicitly not taken for our study.

### Sample collection and preservation

In the course of a study on baboon phylogeography [10], [11], [28] fecal samples from baboons were collected during field surveys from several sites throughout Africa (Figure 1, Tables S1, S2). To ensure that the fecal samples were fresh, of baboon origin, and individually assignable, they were collected directly after baboon individuals were observed to defecate. Samples were preserved following the two-step storage method [40]. Accordingly, fecal samples were preserved in ethanol and 24 hours later transferred to tubes containing silica. Samples were stored at ambient temperature for up to six months before further processing. One additional sample consisted of dry skin tissue from a museum specimen (sample PC3: *Papio cynocephalus*, North-east bank of Lake Rukwa, Tanzania, coll. no. 03-74959, collected in 1902, Humboldt Museum, Berlin, Germany). Blood samples from other papionin genera were obtained from German zoos (*Theropithecus gelada*, Duisburg; *Lophocebus aterrimus*, Berlin; *Cercocebus chrysogaster*, Wuppertal; *Mandrillus sphinx*, Rostock).

### Laboratory methods

DNA from fecal, tissue and blood samples was extracted with the DNeasy and Stool Mini Kit from Qiagen following the supplier's recommendations. For *Rungwecebus*, sequence data from eight loci, including three mitochondrial (12SrRNA, COI, COII), one X chromosomal (Xq13.3), one Y chromosomal (TSPY) and three autosomal loci (α 1,3-GT, LPA, CD4) were available. To amplify respective fragments in baboons and other papionin species, PCR conditions and primers were identical to those used to amplify the loci from *Rungwecebus* [2], [6] and other primates [41]–[44] (Table S3). The Xq13.3 and TSPY sequences were each generated via three overlapping PCR products [2], [6]. The results of the PCR amplifications were checked on agarose gels. PCR products were cleaned with the Qiagen PCR Purification Kit and subsequently sequenced on an ABI 3730xl sequencer using the BigDye Terminator Cycle Sequencing Kit (Applied Biosystems). Sequences were deposited in GenBank (Tables S1, S2). To prevent contamination, laboratory procedures followed recommended, standard protocols [37], [45]–[48]. Specifically, DNA extraction, PCR, PCR purification and sequencing were performed in separate laboratories and repeated after several months, while always only one individual per species was tested. Finally, all PCR reactions were performed with negative (HPLC-purified water) controls.

### Statistical methods

To obtain a comprehensive overview of the phylogenetic position of *Rungwecebus* among papionins, further orthologous sequences from related taxa deposited at GenBank were included in our study (Tables S1, S2). Each of the five nuclear data sets comprised 11 sequences, representing all seven papionin genera and the five recognized baboon species. Each of the three mitochondrial data sets contained 33 sequences, which represent the seven papionin genera including baboons from most of their geographic range. The macaque sequences (*Macaca mulatta* or *M. sylvanus*) were used as an outgroup. Sequences were easily aligned by hand, because few or no indels were present. Due to the low number of polymorphic sites in the nuclear data sets, point mutations, deletions and/or insertions were individually inspected. For phylogenetic tree reconstructions, gaps and poorly aligned positions were manually removed. The sizes of the different alignments and the number of excluded indels are presented in Table S3. Calculations were performed for each locus separately as well as for concatenated nuclear and mitochondrial data sets.

Trees were constructed with maximum-parsimony (MP) and neighbor-joining (NJ) algorithms as implemented in PAUP 4.0b10 [49] as well as with maximum-likelihood (ML) and Bayesian algorithms, using the programs GARLI 0.951 [50] and MrBayes 3.1.2 [51], [52], respectively. For MP analyses, all characters were treated as unordered and equally weighted throughout. A heuristic search was performed with the tree-bisection-reconnection (TBR) algorithm with random addition of sequences. The maximum number of trees was set to 100. NJ, ML and Bayesian trees were constructed with the respective best-fitting models as selected under the Akaike information criterion with MODELTEST 3.7 [53] (Table S3). NJ and ML trees from the concatenated nuclear and mitochondrial data sets were analyzed with the TrN+I and TrN+I+G models, respectively. Bayesian analyses for the combined data set were performed in a partitioned framework, allowing locus-specific parameter estimation. Relative support of internal nodes was performed by bootstrap analyses with 1,000 (MP, NJ) or 500 replications (ML). In GARLI, only the model specifications settings were adjusted according to the respective data set, while all other settings were left at their default value. ML majority-rule consensus trees were calculated in PAUP. For Bayesian analyses, four Monte Carlo Markov Chains with the default temperature of 0.1 were used. Four repetitions were run for 10,000,000 generations with tree and parameter sampling occurring every 100 generations. The first 25% of samples were discarded as burnin, leaving 75,001 trees per run. Posterior probabilities for each split were calculated from the posterior density of trees.

To evaluate the reliability of the depicted phylogenetic position of *Rungwecebus*, alternative tree topologies were evaluated with the KH [7] and SH [8] tests with full optimization and 1,000 bootstrap replications in PAUP. Hypothetical sister group relationships of 1) *Rungwecebus* to *Papio*, *Lophocebus*, *Theropithecus* and *Cercocebus*+*Mandrillus* or 2) that *Rungwecebus* is nested within *Papio* were tested.

Due to the low number of variable sites in the nuclear data set, divergence ages were estimated only for mitochondrial data. Therefore, further outgroup taxa were included in the data set (Table S1). After removal of additional indel positions, the final alignment for divergence age estimations comprised 1,479 bp. A Bayesian MCMC method, which employs a relaxed molecular clock approach [54], as implemented in BEAST v1.4.6 [55], was used. We assumed a relaxed lognormal model of lineage variation and a Yule prior for branching rates. Data were partitioned by manually editing the XML file and by applying the respective best-fitting models and parameters [53]. As calibrations we used the divergence between human and chimpanzee, which has been dated at 6–7 mya [56], [57], the divergence between *Papio* and *Theropithecus*, which is estimated at 3.5–4.0 mya [58], [59 and references therein], and the spilt between hominoids and cercopithecoids, which is estimated at 23 mya [for discussion of fossil data see [60]. Instead of hardbounded calibration points, we used the published dates as a normal distribution prior for the respective node. For C1 (*Pan*/*Homo*) this translates into a normal distribution with a mean of 6.5 mya and a standard deviation of 0.3 mya, for C2 (*Papio/Theropithecus*) into a mean of 3.75 mya and a standard deviation of 0.3 ma (95% credibility interval: 3.0–4.5 mya) and for C3 into a mean of 23.0 mya and a standard deviation of 2.5 ma. Four replicates were run for 10 million generations with tree and parameter sampling occurring every 100 generations. The adequacy of a 10% burnin and convergence of all parameters was assessed by visual inspection of the trace of the parameters across generations using the software TRACER v1.3 [61]. Subsequently, the sampling distributions of four independent replicates were combined and downsampled using the software LogCombiner v1.4.6 and the resulting 10,000 samples summarized and visualized using the software TreeAnnotator v1.4.6 and FigTree v1.1.1 [62]. The first two programs are part of the BEAST package [55].

## Supporting Information

Figure S1 50% majority rule consensus tree (cladogram) based on COI sequences. Numbers on nodes represent bootstrap or posterior probability values (first: MP, second: NJ, third: ML, fourth: Bayesian). Dashes indicate values ≤50%. Red = P. papio, green = P. anubis, grey = P. hamadryas, yellow = P. cynocephalus, brown = P. ursinus. For abbreviations see Fig. 1 and Table S1.
(0.13 MB TIF)

Figure S2 50% majority rule consensus tree (cladogram) based on COII sequences. Numbers on nodes represent bootstrap or posterior probability values (first: MP, second: NJ, third: ML, fourth: Bayesian). Dashes indicate values ≤50%. Red = P. papio, green = P. anubis, grey = P. hamadryas, yellow = P. cynocephalus, brown = P. ursinus. For abbreviations see Fig. 1 and Table S1.
(0.12 MB TIF)

Figure S3 50% majority rule consensus tree (cladogram) based on 12SrRNA sequences. Numbers on nodes represent bootstrap or posterior probability values (first: MP, second: NJ, third: ML, fourth: Bayesian). Dashes indicate values ≤50%. Red = P. papio, green = P. anubis, grey = P. hamadryas, yellow = P. cynocephalus, brown = P. ursinus. For abbreviations see Fig. 1 and Table S1.
(0.09 MB TIF)

Figure S4 50% majority rule consensus tree (cladogram) based on TSPY sequences. Numbers on nodes represent bootstrap or posterior probability values (first: MP, second: NJ, third: ML, fourth: Bayesian). Dashes indicate values ≤50%.
(0.09 MB TIF)

Figure S5 50% majority rule consensus tree (cladogram) based on CD4 sequences. Numbers on nodes represent bootstrap or posterior probability values (first: MP, second: NJ, third: ML, fourth: Bayesian). Dashes indicate values ≤50%.
(0.10 MB TIF)

Figure S6 50% majority rule consensus tree (cladogram) based on α 1,3-GT sequences. Numbers on nodes represent bootstrap or posterior probability values (first: MP, second: NJ, third: ML, fourth: Bayesian). Dashes indicate values ≤50%.
(0.09 MB TIF)

Figure S7 50% majority rule consensus tree (cladogram) based on LPA sequences. Numbers on nodes represent bootstrap or posterior probability values (first: MP, second: NJ, third: ML, fourth: Bayesian).
(0.09 MB TIF)

Figure S8 50% majority rule consensus tree (cladogram) based on Xq13.3 sequences. Numbers on nodes represent bootstrap or posterior probability values (first: MP, second: NJ, third: ML, fourth: Bayesian). Dashes indicate values ≤50%.
(0.09 MB TIF)

Table S1 Origin of analyzed samples for mitochondrial DNA studies and their GenBank accession numbers (* marked samples were used also for the analysis of nuclear loci).
(0.38 MB DOC)

Table S2 Species and GenBank accession numbers for nuclear DNA studies. For abbreviation and origin of baboon samples see Table S1.
(0.04 MB DOC)

Table S3 Detailed information about primers, PCR conditions, applied substitution models, sequence length and number of polymorphic sites.
(0.04 MB DOC)

Table S4 Mutational events including point mutations, deletions and insertions in the five nuclear loci.
(0.25 MB DOC)
