# Supplementary figures and images for: Is the New Primate Genus Rungwecebus a Baboon?
Source: PLoS One. 2009 Mar 19;4(3):e4859. doi: 10.1371/journal.pone.0004859 (PMC2654078; doi:10.1371/journal.pone.0004859)

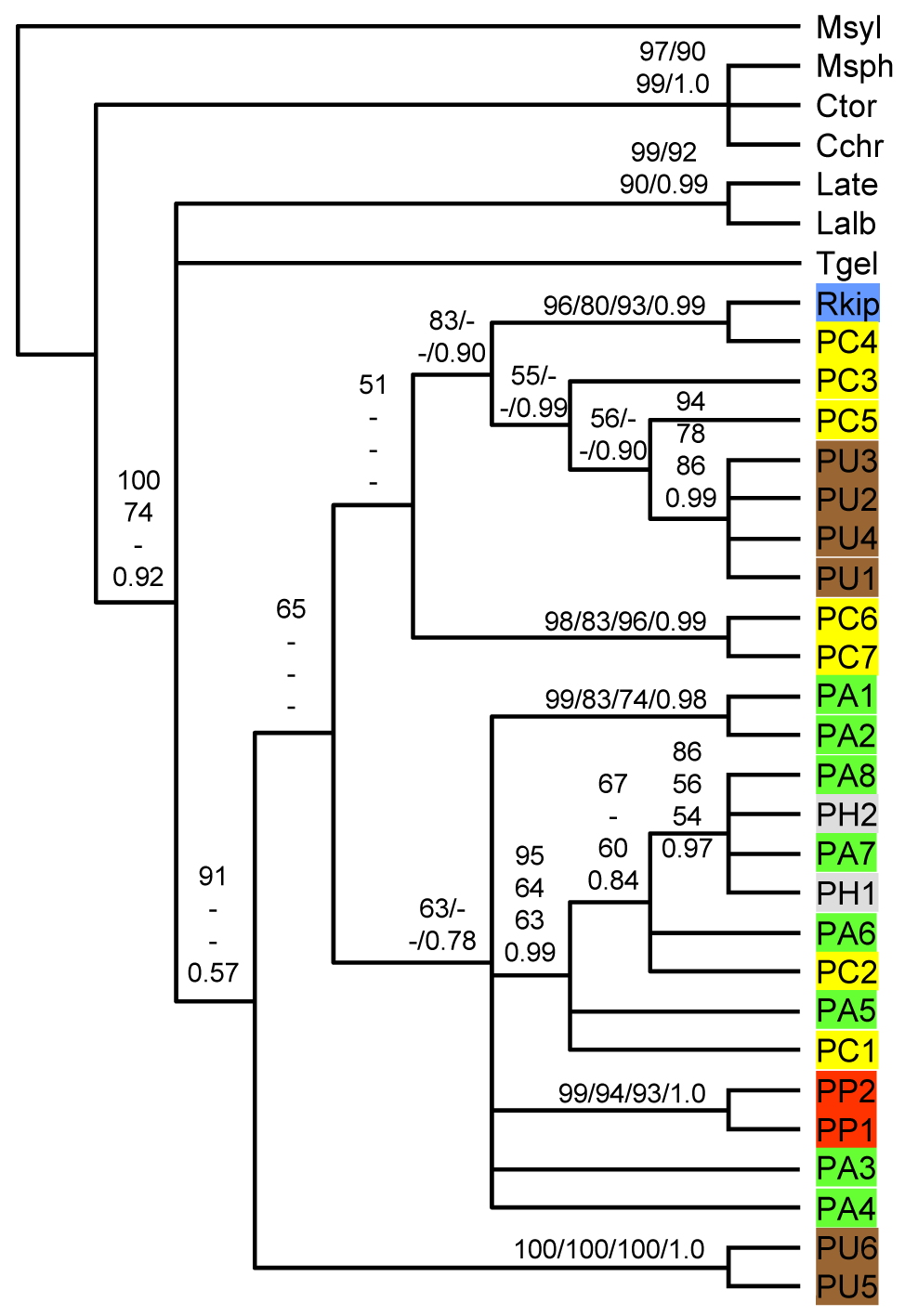

Supplement: Figure S1 — 50% majority rule consensus tree (cladogram) based on COI sequences. Numbers on nodes represent bootstrap or posterior probability values (first: MP, second: NJ, third: ML, fourth: Bayesian). Dashes indicate values ≤50%. Red = P. papio, green = P. anubis, grey = P. hamadryas, yellow = P. cynocephalus, brown = P. ursinus. For abbreviations see Fig. 1 and Table S1. (0.13 MB TIF) [file pone.0004859.s001.tif]

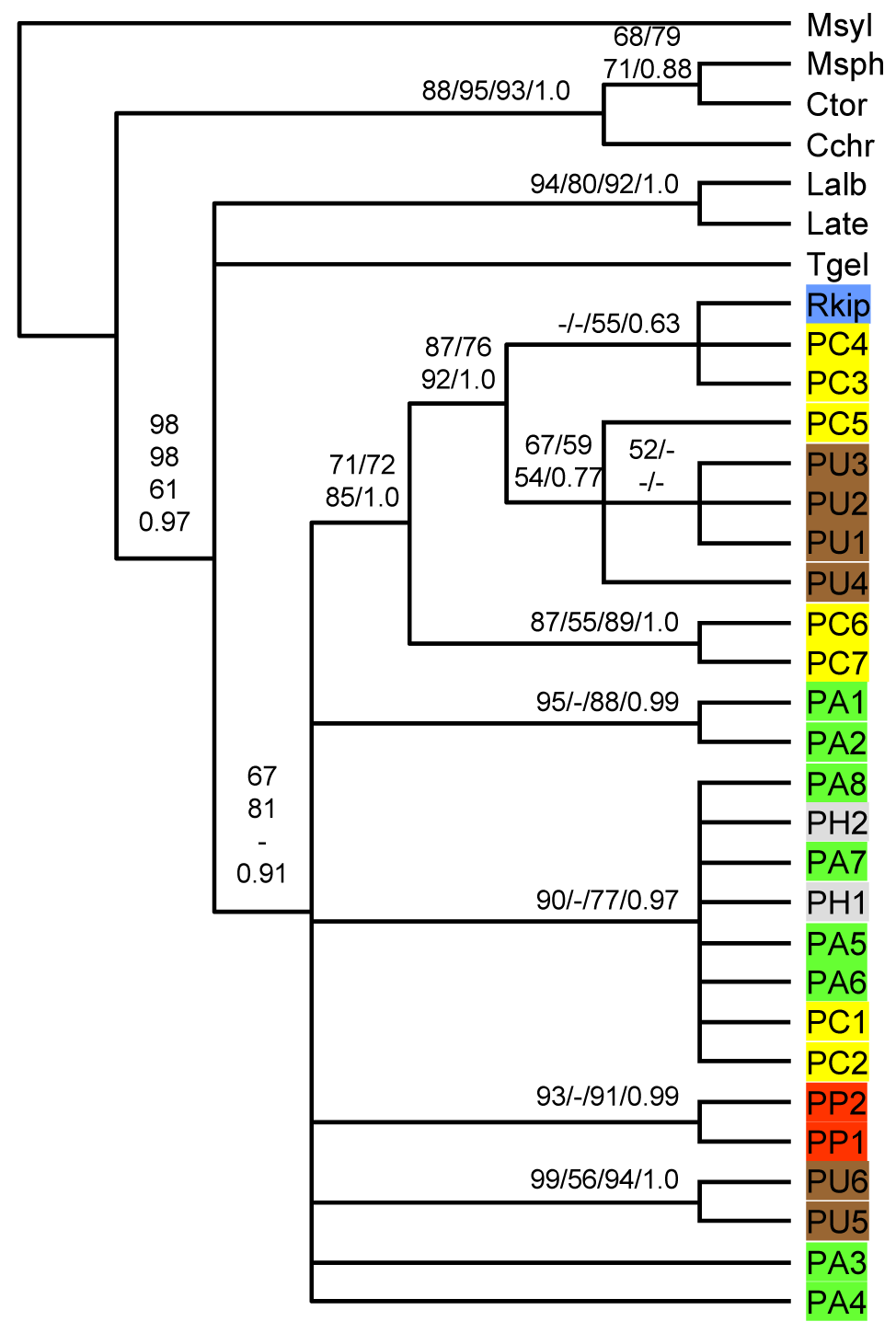

Supplement: Figure S2 — 50% majority rule consensus tree (cladogram) based on COII sequences. Numbers on nodes represent bootstrap or posterior probability values (first: MP, second: NJ, third: ML, fourth: Bayesian). Dashes indicate values ≤50%. Red = P. papio, green = P. anubis, grey = P. hamadryas, yellow = P. cynocephalus, brown = P. ursinus. For abbreviations see Fig. 1 and Table S1. (0.12 MB TIF) [file pone.0004859.s002.tif]

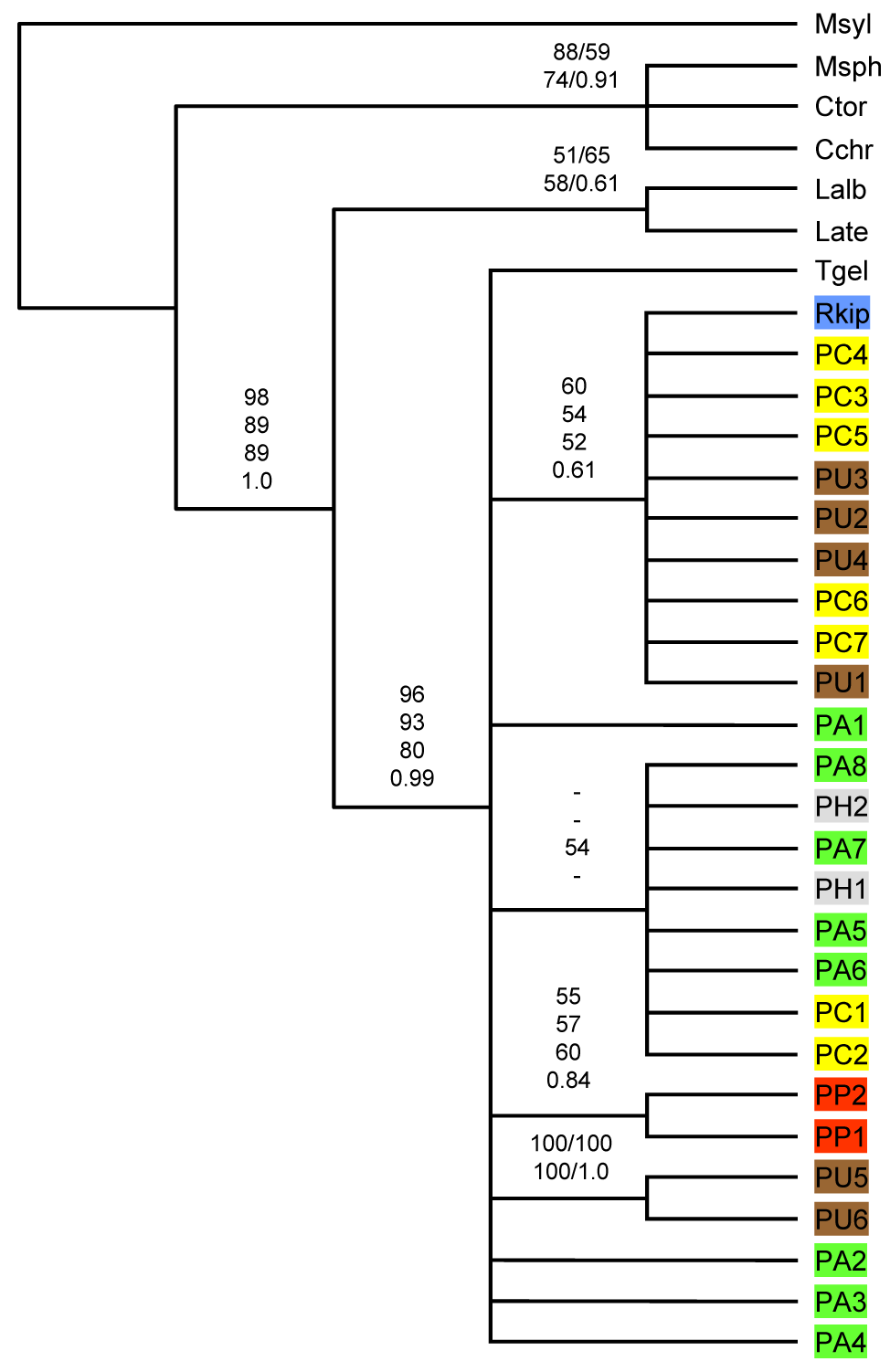

Supplement: Figure S3 — 50% majority rule consensus tree (cladogram) based on 12SrRNA sequences. Numbers on nodes represent bootstrap or posterior probability values (first: MP, second: NJ, third: ML, fourth: Bayesian). Dashes indicate values ≤50%. Red = P. papio, green = P. anubis, grey = P. hamadryas, yellow = P. cynocephalus, brown = P. ursinus. For abbreviations see Fig. 1 and Table S1. (0.09 MB TIF) [file pone.0004859.s003.tif]

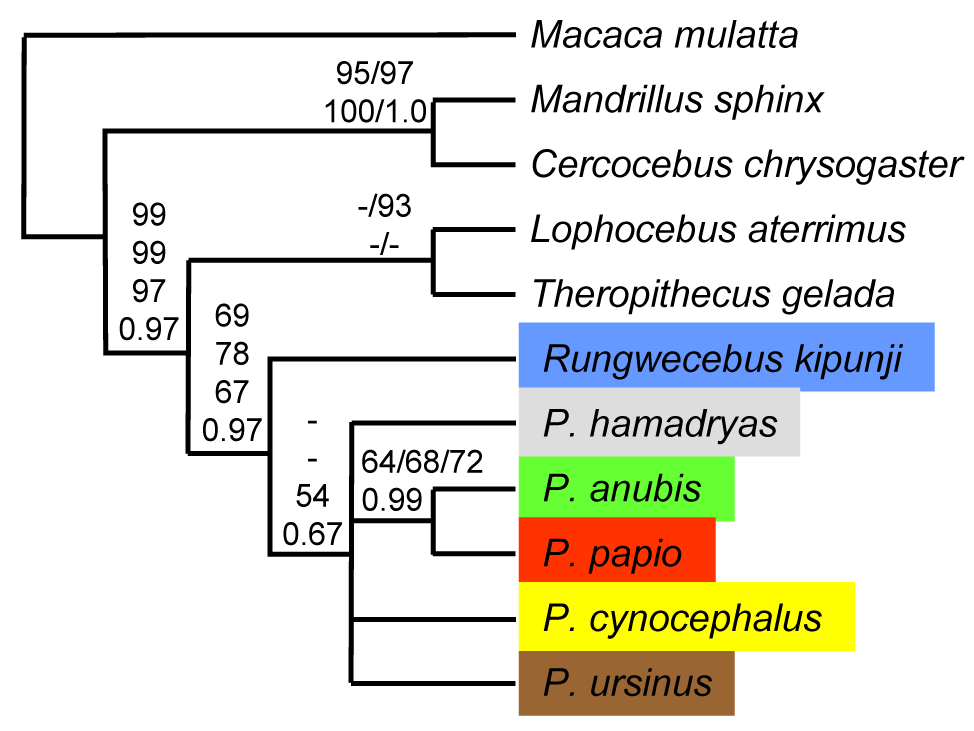

Supplement: Figure S4 — 50% majority rule consensus tree (cladogram) based on TSPY sequences. Numbers on nodes represent bootstrap or posterior probability values (first: MP, second: NJ, third: ML, fourth: Bayesian). Dashes indicate values ≤50%. (0.09 MB TIF) [file pone.0004859.s004.tif]

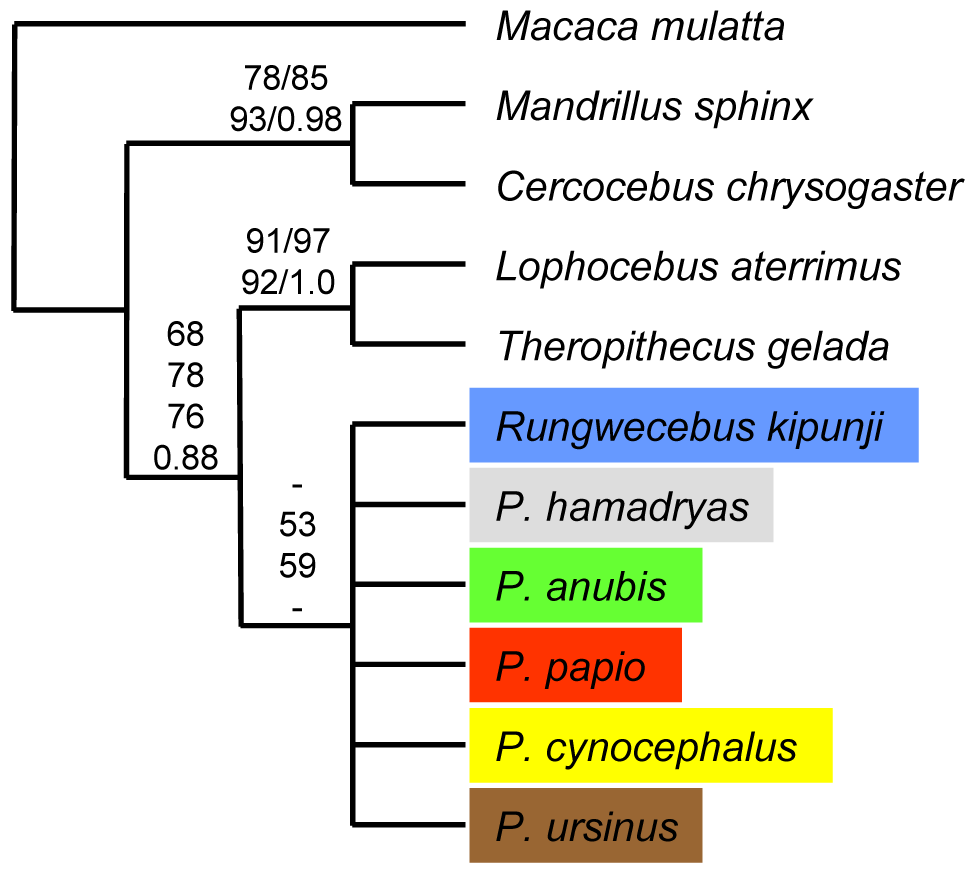

Supplement: Figure S5 — 50% majority rule consensus tree (cladogram) based on CD4 sequences. Numbers on nodes represent bootstrap or posterior probability values (first: MP, second: NJ, third: ML, fourth: Bayesian). Dashes indicate values ≤50%. (0.10 MB TIF) [file pone.0004859.s005.tif]

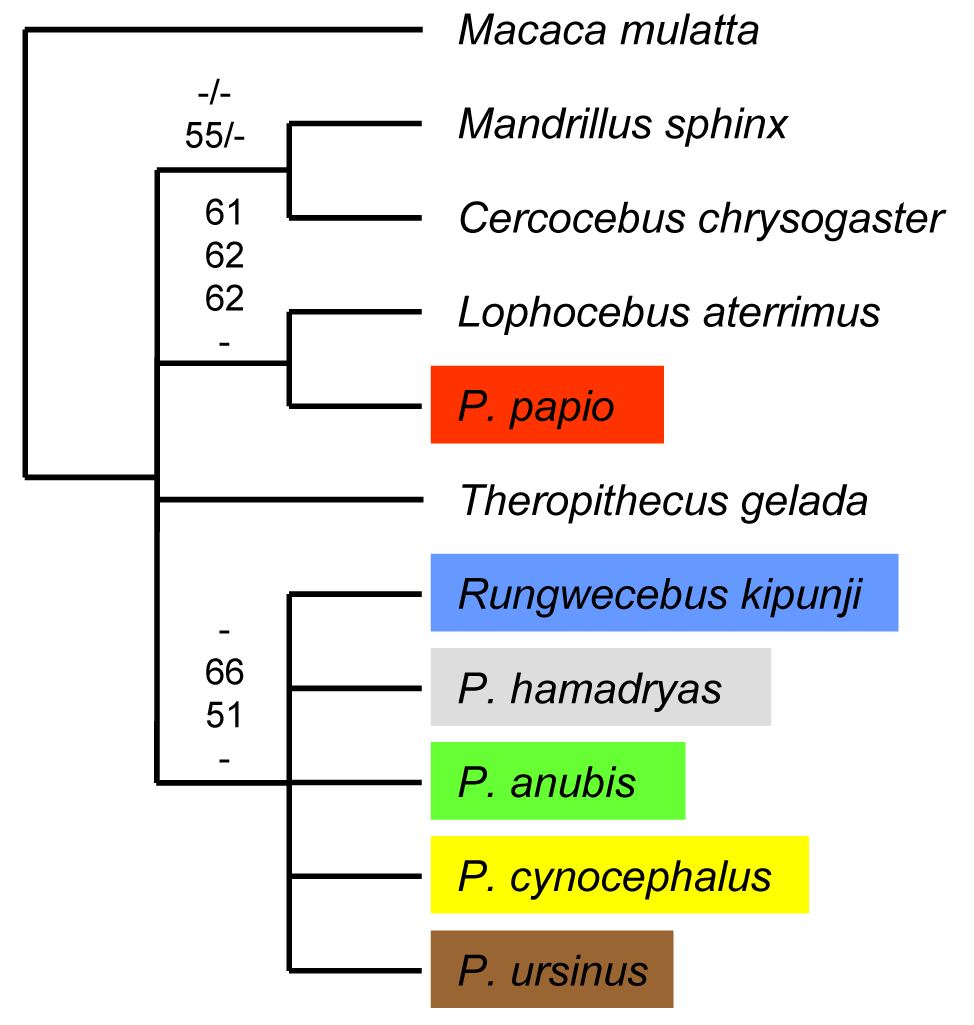

Supplement: Figure S6 — 50% majority rule consensus tree (cladogram) based on α 1,3-GT sequences. Numbers on nodes represent bootstrap or posterior probability values (first: MP, second: NJ, third: ML, fourth: Bayesian). Dashes indicate values ≤50%. (0.09 MB TIF) [file pone.0004859.s006.tif]

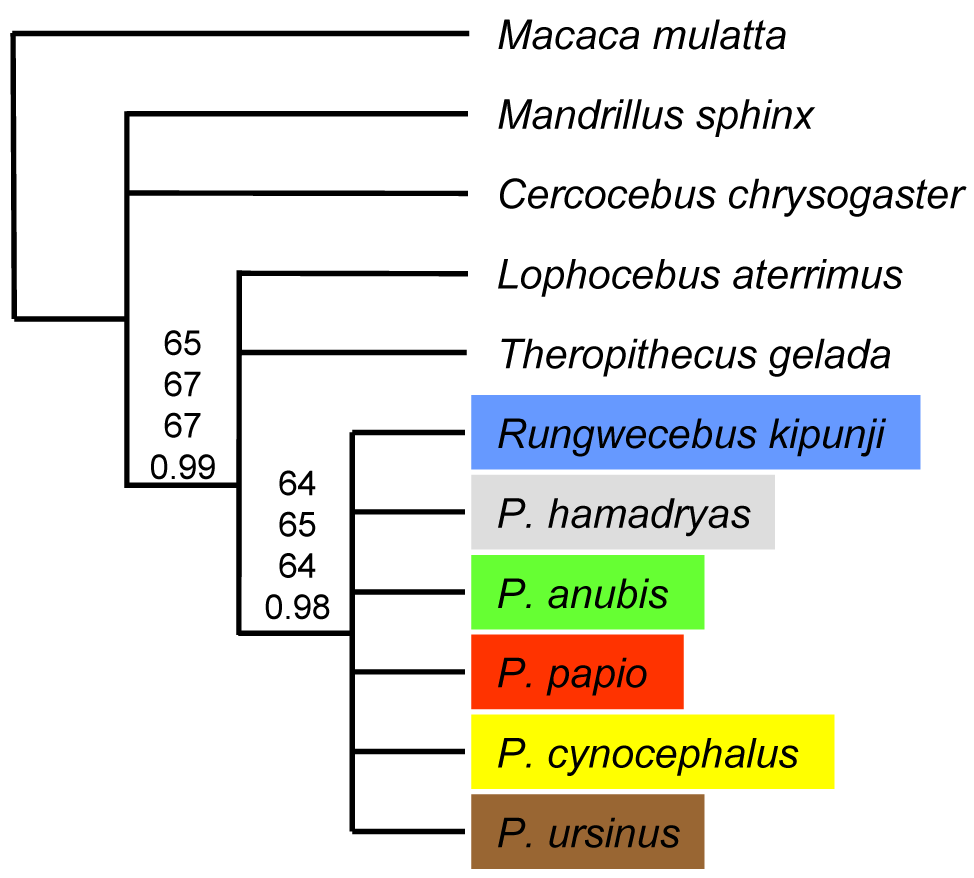

Supplement: Figure S7 — 50% majority rule consensus tree (cladogram) based on LPA sequences. Numbers on nodes represent bootstrap or posterior probability values (first: MP, second: NJ, third: ML, fourth: Bayesian). (0.09 MB TIF) [file pone.0004859.s007.tif]

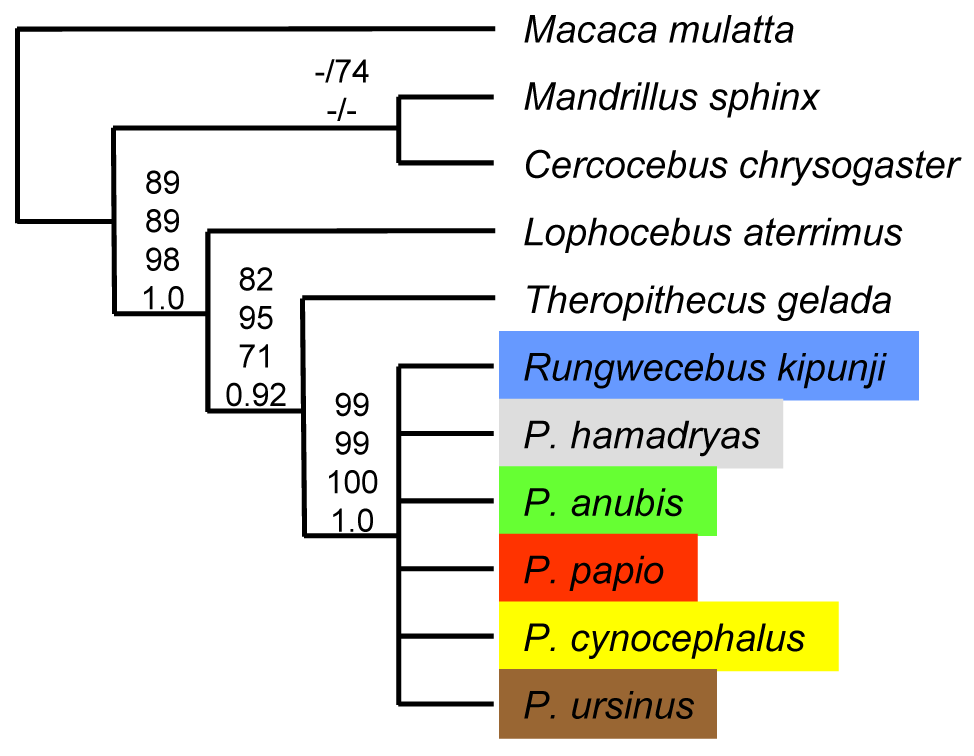

Supplement: Figure S8 — 50% majority rule consensus tree (cladogram) based on Xq13.3 sequences. Numbers on nodes represent bootstrap or posterior probability values (first: MP, second: NJ, third: ML, fourth: Bayesian). Dashes indicate values ≤50%. (0.09 MB TIF) [file pone.0004859.s008.tif]
