# Supplementary material for: Is the New Primate Genus Rungwecebus a Baboon?
Source: PLoS One. 2009 Mar 19;4(3):e4859. doi: 10.1371/journal.pone.0004859 (PMC2654078; doi:10.1371/journal.pone.0004859)
Supplement: Table S1 — Origin of analyzed samples for mitochondrial DNA studies and their GenBank accession numbers (* marked samples were used also for the analysis of nuclear loci). (0.38 MB DOC) [file pone.0004859.s009.doc]

**Table S1.** Origin of analyzed samples for mitochondrial DNA studies and their GenBank accession numbers (* marked samples were used also for the analysis of nuclear loci).

| ID | Taxon | Country | Site | Longitude/  Latitude | 12SrRNA | COI | COII |
| --- | --- | --- | --- | --- | --- | --- | --- |
| RKI | *Rungwecebus kipunjii* | Tanzania |  |  | DQ375756 | DQ381473 | DQ381471 |
| PP1* | *Papio papio* | Senegal | Niokolo Koba | -12.72090  13.07467 | FJ750680 | FJ750652 | EU293078 |
| PP2 | *Papio papio* | Guinea | Haute Niger | -10.31542  10.54267 | FJ750681 | FJ750653 | EU293077 |
| PA1 | *Papio anubis* | Ivory Coast | Komoé | -3.79000  8.80000 | FJ750682 | FJ750654 | FJ750634 |
| PA2 | *Papio anubis* | Nigeria | Lumma | 4.26420  10.31810 | FJ750683 | FJ750655 | FJ750635 |
| PA3 | *Papio anubis* | Nigeria | Chigwa | 7.81670  9.35000 | FJ750684 | FJ750656 | FJ750636 |
| PA4 | *Papio anubis* | Nigeria | Gashaka Gumti | 11.50000  7.35000 | FJ750685 | FJ750657 | FJ750637 |
| PA5 | *Papio anubis* | Uganda | Kibale | 30.40000  0.48333 | FJ750686 | FJ750658 | FJ750638 |
| PA6* | *Papio anubis* | Kenya | Segera | 36.01581  0.25338 | FJ750687 | FJ750659 | FJ750639 |
| PA7 | *Papio anubis* | Ethiopia | Managasha | 38.57125  8.96838 | FJ750688 | FJ750660 | EU293069 |
| PA8 | *Papio anubis* | Eritrea | Ruba Grivet | 36.76018  14.88322 | FJ750689 | FJ750661 | EU293068 |
| PH1* | *Papio hamadryas* | Eritrea | Afabet | 38.74958  16.12017 | FJ750690 | FJ750662 | FJ750640 |
| PH2 | *Papio hamadryas* | Ethiopia | Gerba Luku | 41.53400  9.58740 | FJ750691 | FJ750663 | EU293066 |
| PC1 | *Papio cynocephalus* | Kenya | Diani Beach | 39.55000  -4.32000 | FJ750692 | FJ750664 | FJ75041 |
| PC2 | *Papio cynocephalus* | Kenya | Amboseli | 37.39000  -2.29000 | FJ750693 | FJ750665 | EU293071 |
| PC3* | *Papio cynocephalus* | Tanzania | Lake Rukwa | 32.15517  -7.58297 | FJ750694 | FJ750666 | EU293073 |
| PC4 | *Papio cynocephalus* | Malawi | Michiru Mts | 34.91667  -15.75000 | FJ750695 | FJ750667 | EU293072 |
| PC5 | *Papio cynocephalus* | Zambia | South Luangwa | 31.63793  -13.26840 | FJ750696 | FJ750668 | EU293074 |
| PC6 | *Papio cynocephalus* | Zambia | Kafue North | 26.53577  -14.96779 | FJ750697 | FJ750669 | FJ750642 |
| PC7 | *Papio cynocephalus* | Zambia | Shiwa N’gandu | 31.73892  -11.19677 | FJ750698 | FJ750670 | FJ750643 |
| PU1 | *Papio ursinus* | Zambia | Kafue Middle | 25.97031  -15.87178 | FJ750699 | FJ750671 | FJ750644 |
| PU2 | *Papio ursinus* | Zimbabwe | Bindura | 31.39892  -17.21172 | FJ750700 | FJ750672 | FJ750645 |
| PU3 | *Papio ursinus* | Namibia | Waterberg Plateau | 17.24221  -20.50450 | FJ750701 | FJ750673 | FJ750646 |
| PU4 | *Papio ursinus* | South Africa | Ithala | 31.26667  -27.53333 | FJ750702 | FJ750674 | FJ750647 |
| PU5 | *Papio ursinus* | Namibia | Namib Spreetshoogte | 16.20555  -23.64758 | FJ750703 | FJ750675 | FJ750648 |
| PU6* | *Papio ursinus* | South Africa | DeHoop | 20.40658  -34.45621 | FJ750704 | FJ750676 | EU293075 |
| Tgel | *Theropithecus gelada* | - | - | - | AF424945 | AY972675 | EU293079 |
| Lalb | *Lophocebus albigena* | - | - | - | AY665614 | AY972693 | AY686130 |
| Late | *Lophocebus aterrimus* | - | - | - | FJ750705 | FJ750677 | FJ750649 |
| Ctor | *Cercocebus torquatus* | - | - | - | L35204 | EU179511 | AY686135 |
| Cchr | *Cercocebus chrysogaster* | - | - | - | FJ750706 | FJ750678 | FJ750650 |
| Msph | *Mandrillus sphinx* | - | - | - | FJ750707 | FJ750679 | FJ750651 |
| Msyl | *Macaca sylvanus* |  |  |  | AJ309865 | AJ309865 | AJ309865 |
| **Divergence age estimation** | | | | | | | |
|  | *Macaca*  *mulatta* |  |  |  | AY612638 | AY612638 | AY612638 |
|  | *Cholorocebus aethiops* |  |  |  | NC_00700 | NC_00700 | NC_00700 |
|  | *Colobus*  *guereza* |  |  |  | NC_00690 | NC_00690 | NC_00690 |
|  | *Homo*  *sapiens* |  |  |  | AY339522 | AY339522 | AY339522 |
|  | *Pan*  *troglodytes* |  |  |  | D38113 | D38113 | D38113 |
|  | *Cebus*  *olivaceus* | - | - | - | AJ309866 | AJ309866 | AJ309866 |
