# Supplementary material for: Is the New Primate Genus Rungwecebus a Baboon?
Source: PLoS One. 2009 Mar 19;4(3):e4859. doi: 10.1371/journal.pone.0004859 (PMC2654078; doi:10.1371/journal.pone.0004859)
Supplement: Table S2 — Species and GenBank accession numbers for nuclear DNA studies. For abbreviation and origin of baboon samples see Table S1. (0.04 MB DOC) [file pone.0004859.s010.doc]

**Table S2.** Species and GenBank accession numbers for nuclear DNA studies. For abbreviation and origin of baboon samples see Table S1.

| **Taxon** | **CD4** | **LPA** | **α 1,3-GT** | **Xq13.3** | **TSPY** |
| --- | --- | --- | --- | --- | --- |
| *Rungwecebus kipunjii* | EU600174 | EU600172 | DQ381470 | EU600173 | DQ381472 |
| *Papio hamadryas* (PH1) | FJ750589 | FJ750598 | FJ750607 | FJ750616 | FJ750625 |
| *Papio papio* (PP1) | FJ750590 | FJ750599 | FJ750608 | FJ750617 | FJ750626 |
| *Papio anubis* (PA6) | FJ750591 | FJ750600 | FJ750609 | FJ750618 | FJ750627 |
| *Papio cynocephalus* (PC3) | FJ750592 | FJ750601 | FJ750610 | FJ750619 | FJ750628 |
| *Papio ursinus* (PU6) | FJ750593 | FJ750602 | FJ750611 | FJ750620 | FJ750629 |
| *Theropithecus gelada* | FJ750594 | FJ750603 | FJ750612 | FJ750621 | FJ750630 |
| *Lophocebus aterrimus* | FJ750595 | FJ750604 | FJ750613 | FJ750622 | FJ750631 |
| *Mandrillus sphinx* | FJ750596 | FJ750605 | FJ750614 | FJ750623 | FJ750632 |
| *Cercocebus chrysogaster* | FJ750597 | FJ750606 | FJ750615 | FJ750624 | FJ750633 |
| *Macaca mulatta* | AF057385 | AY192772 | AY026237 | AY899239 | AF425276 |
