# Supplementary material for: Is the New Primate Genus Rungwecebus a Baboon?
Source: PLoS One. 2009 Mar 19;4(3):e4859. doi: 10.1371/journal.pone.0004859 (PMC2654078; doi:10.1371/journal.pone.0004859)
Supplement: Table S3 — Detailed information about primers, PCR conditions, applied substitution models, sequence length and number of polymorphic sites. (0.04 MB DOC) [file pone.0004859.s011.doc]

**Table S3.** Detailed information about primers, PCR conditions, applied substitution models, sequence length and number of polymorphic sites.

| **Locus** | **Primer + PCR conditions** | **Substitution model** | **Length / indels removed** | **Polymorphic sites / parsimony-informative sites** |
| --- | --- | --- | --- | --- |
| COI | [37] | TrN + I + G | 600 / - | 195 / 144 |
| COII | [38] | TrN + G | 500 / - | 138 / 104 |
| 12SrRNA | [35] | TrN + I + G | 394 / 8 | 62 / 41 |
| mitochondrial DNA combined | - | TrN + I + G | 1494 / 8 | 395 / 289 |
| TSPY | [6,36] | TrN + I | 1592 / - | 62 / 17 |
| CD4 | [36] | TrN + I | 580 / - | 16 / 6 |
| α 1,3-GT | [36] | TrN + I | 486 / 9 | 9 / 5 |
| LPA | [6] | TrN | 578 / 2 | 12 / 2 |
| Xq13.3 | [6] | GTR | 1273 / 12 | 52 / 11 |
| nuclear DNA combined | - | TrN + I | 4509 / 23 | 151 / 41 |
