# Supplementary material for: Is the New Primate Genus Rungwecebus a Baboon?
Source: PLoS One. 2009 Mar 19;4(3):e4859. doi: 10.1371/journal.pone.0004859 (PMC2654078; doi:10.1371/journal.pone.0004859)
Supplement: Table S4 — Mutational events including point mutations, deletions and insertions in the five nuclear loci. (0.25 MB DOC) [file pone.0004859.s012.doc]

**Table S4.** Mutational events including point mutations, deletions and insertions in the 5 nuclear loci (CD4; LPA; α 1,3-GT; Xq13.3; TSPY).

| **position** | **locus** | ***Rungwecebus*** | ***Papio*** | ***Theropithecus*** | ***Lophocebus*** | ***Mandrillus*** | ***Cercocebus*** |
| --- | --- | --- | --- | --- | --- | --- | --- |
| 64 | CD4 | C | C | C | C | T | T |
| 75 | CD4 | C | C | G | C | C | C |
| 102 | CD4 | G | G | G | G | A | A |
| 144 | CD4 | T | T | C | C | C | C |
| 152 | CD4 | G | G | A | G | G | G |
| 179 | CD4 | G | G | G | G | A | G |
| 191 | CD4 | G | G | A | A | G | G |
| 230 | CD4 | A | A | G | G | A | A |
| 282 | CD4 | A | A | A | A | A | G |
| 341-343 | CD4 | GGA | GGA/- - - | GGA | GGA | GGA | GGA |
| 455 | CD4 | C | C | C | T | C | C |
| 520 | CD4 | C | C | C | C | G | G |
| 568 | CD4 | G | G | G | T | G | G |
|  | | | | | | | |
| **position** | **locus** | ***Rungwecebus*** | ***Papio*** | ***Theropithecus*** | ***Lophocebus*** | ***Mandrillus*** | ***Cercocebus*** |
| 66 | LPA | A | A | G | G | G | G |
| 107 | LPA | G | G | G | G | A | G |
| 163 | LPA | A | A | A | A | A | G |
| 199 | LPA | G | G | G | G | T | T |
| 291 | LPA | G | G | G | G | A | G |
|  | | | | | | | |
| **position** | **locus** | ***Rungwecebus*** | ***Papio*** | ***Theropithecus*** | ***Lophocebus*** | ***Mandrillus*** | ***Cercocebus*** |
| 47 | α 1,3-GT | C | C/A | C | C | C | C |
| 89 | α 1,3-GT | T | T | T | C | T/C | T |
| 124-132 | α 1,3-GT | GAGGTTGAC | GAGGTTGAC | GAGGTCGAC | GAGGTCTAC | - - - - - - - - - | GAGGTCGAC |
| 242 | α 1,3-GT | T | T | A | A | A | A |
| 284 | α 1,3-GT | C | C | C | T | T | T |
| 439 | α 1,3-GT | A | C/A | A | C | A | A |
| 447 | α 1,3-GT | C | C/T | C | T | C | C |
| 453 | α 1,3-GT | C | C/G | C | G | C | C |
|  | | | | | | | |
| **position** | **locus** | ***Rungwecebus*** | ***Papio*** | ***Theropithecus*** | ***Lophocebus*** | ***Mandrillus*** | ***Cercocebus*** |
| 20 | Xq13.3 | C | C | T | T | T | T |
| 89 | Xq13.3 | A | A | C | G | G | G |
| 142 | Xq13.3 | G | G | G | G | C | G |
| 153-158 | Xq13.3 | TACACT | TACACT | TACACT | TACACT | - - - - - - | TACACT |
| 181 | Xq13.3 | A | A | A | A | A | G |
| 199 | Xq13.3 | A | A | A | A | A | G |
| 267 | Xq13.3 | T | T | T | T | C | T |
| 287 | Xq13.3 | T | T | T | T | A | T |
| 296 | Xq13.3 | C | C | C | G | C | C |
| 365 | Xq13.3 | A | A | A | A | A | G |
| 367 | Xq13.3 | G | G | G | G | G | C |
| 387 | Xq13.3 | C | C | C | C | T | C |
| 417 | Xq13.3 | T | T | G | T | T | T |
| 422 | Xq13.3 | A | A | A | A | G | A |
| 432 | Xq13.3 | T | T | T | T | C | C |
| 456-458 | Xq13.3 | TAA | TAA | - - - | - -A | T-A | - -A |
| 464 | Xq13.3 | T | T | T | T | G | G |
| 478 | Xq13.3 | T | T | T | T | T | C |
| 519 | Xq13.3 | T | T/A | T | T | T | T |
| 585 | Xq13.3 | C | C | C | C | C | G |
| 586 | Xq13.3 | A | A | A | A | A | G |
| 591 | Xq13.3 | G | G | G | A | G | G |
| 592 | Xq13.3 | T | T | T | T | T | A |
| 608 | Xq13.3 | C | C | C | C | C | T |
| 638 | Xq13.3 | T | T | T | C | T | T |
| 681 | Xq13.3 | T | T | T | T | T | C |
| 731 | Xq13.3 | A | A | A | A | G | A |
| 832 | Xq13.3 | G | G/- | G | G | G | G |
| 842 | Xq13.3 | A | A | A | A | G | A |
| 864 | Xq13.3 | C | C | C | C | C | - |
| 967 | Xq13.3 | T | T | A | A | A | A |
| 983 | Xq13.3 | G | G | G | - | G | G |
| 994 | Xq13.3 | A | A | A | T | A | A |
| 1028 | Xq13.3 | A | A | A | A | G | G |
| 1037 | Xq13.3 | C | C | C | T | C | C |
| 1066 | Xq13.3 | G | G | G | G | G | C |
| 1067 | Xq13.3 | A | A | A | A | C | C |
| 1097 | Xq13.3 | G | G | G | A | G | G |
| 1127 | Xq13.3 | A | A | G | G | G | G |
| 1163 | Xq13.3 | T | T | T | T | A | T |
| 1195 | Xq13.3 | G | G | C | G | G | G |
| 1196 | Xq13.3 | C | C | C | T | T | T |
| 1208 | Xq13.3 | G | G | G | C | G | G |
| 1217 | Xq13.3 | C | C | C | T | C | C |
| 1268 | Xq13.3 | C | C | T | T | T | T |
|  | | | | | | | |
| **position** | **locus** | ***Rungwecebus*** | ***Papio*** | ***Theropithecus*** | ***Lophocebus*** | ***Mandrillus*** | ***Cercocebus*** |
| 12 | TSPY | A | A | A | A | G | A |
| 22 | TSPY | T | T | T | C | T | T |
| 73 | TSPY | A | A | A | A | C | A |
| 92 | TSPY | G | G | C | G | G | G |
| 133 | TSPY | A | A | A | A | G | A |
| 138 | TSPY | T | T | T | T | G | T |
| 172 | TSPY | A | A | G | A | A | A |
| 181 | TSPY | G | G | G | G | A | A |
| 193 | TSPY | T | T | G | T | T | T |
| 260 | TSPY | C | C/T | T | T | T | T |
| 270 | TSPY | G | G | G | G | A | A |
| 298 | TSPY | A | A | A | A | T | A |
| 312 | TSPY | T | T | T | C | T | T |
| 387 | TSPY | T | T | T | T | A | A |
| 398 | TSPY | T | T/C | T | T | T | T |
| 472 | TSPY | C | C | C | C | G | G |
| 500 | TSPY | A | A | A | G | A | A |
| 534 | TSPY | T | G | T | T | T | T |
| 555 | TSPY | A | A | A | A | G | A |
| 582 | TSPY | G | G | G | G | A | G |
| 589 | TSPY | T | T | T | T | T | C |
| 681 | TSPY | G | T | T | T | T | T |
| 694 | TSPY | T | T | C | T | T | T |
| 721 | TSPY | C | C | C | C | T | T |
| 729 | TSPY | T | T | T | T | C | C |
| 762 | TSPY | T | T | T | T | C | C |
| 778 | TSPY | A | A | A | A | G | A |
| 857 | TSPY | A | A | A | A | A | G |
| 935 | TSPY | A | A | A | A | A | T |
| 943 | TSPY | A | A | A | A | A | C |
| 1029 | TSPY | A | A | A | A | A | C |
| 1030 | TSPY | A | A | A | A | A | G |
| 1082 | TSPY | A | A | A | A | G | G |
| 1084 | TSPY | A | A | G | A | A | A |
| 1136 | TSPY | A | A | A | A | G | G |
| 1159 | TSPY | T | T | T | T | T | C |
| 1182 | TSPY | C | C | C | C | G | G |
| 1244 | TSPY | A | A | A | G | A | A |
| 1306 | TSPY | T | T | C | T | T | T |
| 1333 | TSPY | T | T | T | T | C | T |
| 1434 | TSPY | A | A | A | G | A | A |
| 1466 | TSPY | T | T | C | C | C | C |
| 1469 | TSPY | A | A | A | G | A | A |
| 1510 | TSPY | A | A | A | A | A | G |
| 1526 | TSPY | C | C | C | C | T | T |
| 1532 | TSPY | A | A | A | A/G | A | A |
| 1534 | TSPY | G | G | G | A | G | G |
| 1539 | TSPY | T | T | T | T | C | T |
| 1567 | TSPY | A | A | A | A | A | G |
| 1584 | TSPY | C | C | C | C | G | C |
